# High Proportion of HIV-HCV Coinfected Patients with Advanced Liver Fibrosis Requiring Hepatitis C Treatment in Haiphong, Northern Vietnam (ANRS 12262)

**DOI:** 10.1371/journal.pone.0153744

**Published:** 2016-05-05

**Authors:** Tam Nguyen Truong, Didier Laureillard, Karine Lacombe, Huong Duong Thi, Phuc Pham Thi Hanh, Lien Truong Thi Xuan, Nga Chu Thi, Anh Luong Que, Vinh Vu Hai, Nicolas Nagot, Edouard Tuaillon, Stéphanie Dominguez, Maud Lemoine

**Affiliations:** 1 University of Medicine Pham Ngoc Thach, Ho Chi Minh City, Vietnam; 2 INSERM U1058 "Pathogenesis and Control of Chronic Infections", Montpellier, France; 3 Department of Infectious and Tropical Diseases, Caremeau University Hospital, Nîmes, France; 4 Department of Infectious and Tropical Diseases, Saint-Antoine Hospital, AP-HP, Paris, France; 5 Sorbonne Universités, UPMC Université Paris 06, UMR_S 1136, Institut Pierre Louis d’Epidémiologie et de Santé Publique, Paris, France; 6 Department of Public Health, University of Medicine and Pharmacy, Hai Phong, Vietnam; 7 Department of Infectious and Tropical Diseases, Viet Tiep Hospital, Hai Phong, Vietnam; 8 Pasteur Institute, Ho Chi Minh City, Vietnam; 9 Department of virology, Viet Tiep hospital, Haiphong, Vietnam; 10 Department of epidemiology, University Hospital, Montpellier, France; 11 Department of bacteriology and virology, University Hospital, Montpellier, France; 12 Department of clinical immunology, Henri Mondor Hospital, AP-HP, Créteil, France; 13 Department of Hepatology, St Mary’s Hospital, Imperial College, London, United Kingdom; CRCL-INSERM, FRANCE

## Abstract

**Rationale and Aims:**

Screening and treatment for chronic hepatitis C are very limited in Vietnam and clinical data on HCV-related liver disease in HIV-coinfected people are almost inexistent. This study aimed to assess the severity of liver fibrosis and its risk factors in HIV-HCV coinfected patients in Haiphong, Northern Vietnam.

**Methods:**

A cross-sectional study was conducted at a HIV outpatient clinic. Consecutive HIV treated adults with positive HCV serology completed a standardised epidemiological questionnaire and had a comprehensive liver assessment including hepatic elastography (Fibroscan^®^, Echosens).

**Results:**

From February to March 2014, 104 HIV-HCV coinfected patients receiving antiretroviral therapy (ART) were prospectively enrolled (99 males, median age: 35.8 (32.7–39.6) years, median CD4 count: 504 (361–624) /mm^3^. Of them, 93 (89.4%) had detectable HCV RNA (median 6.19 (4.95–6.83 Log_10_ IU/mL). Patients were mainly infected with genotypes 1a/1b (69%) and genotypes 6a/6e (26%). Forty-three patients (41.3%) had fibrosis ≥F2 including 24 patients (23.1%) with extensive fibrosis (F3) and/or cirrhosis (F4). In univariate analysis, excessive alcohol consumption, estimated time duration from HCV infection, nevirapine and lopinavir-based ARV regimen and CD4 nadir were associated factors of extensive fibrosis/cirrhosis. Alcohol abuse was the only independent factor of extensive fibrosis in multivariate analysis. Using Fibroscan^®^ as a gold standard, the high thresholds of AST-to-platelet ratio index (APRI) and fibrosis-4 score (FIB-4) had very good performances for the diagnosis of extensive fibrosis/cirrhosis (Se: 90 and 100%, Sp:84 and 81%, AUROCs = 0.93, 95%CI: 0.86–0.99 and 0.96 (0.92–0.99), respectively).

**Conclusion:**

In this study, nearly 25% of HIV-HCV coinfected patients successfully treated with ART have extensive fibrosis or cirrhosis, and therefore require urgently HCV treatment.

## Introduction

Over the last decade, access to HIV/AIDS care and antiretroviral therapy (ART) has been greatly improved in resource-limited countries and the number of AIDS-related deaths is now steadily decreasing [1]. Hepatitis C virus (HCV) infection, which is a major public health problem globally [2] is frequently observed in HIV-infected individuals and has emerged as a leading cause of morbidity and mortality in this population [3].

In Vietnam, HCV seroprevalence has been reported between 1 to 6% of the general population [4] and is dramatically higher (up to 90%) in people who inject drug (PWID) [4–10]. The HIV epidemic is concentrated among high risk populations, such as female sex workers (prevalence 2.6%), men who have sex with men (3.7%) and PWID (10.3%) [11]. Despite huge efforts to control the national HIV epidemic among PWID by scaling up ART, needle & syringe exchange programmes and opioid substitution treatment, PWID still represent the vast majority of people living with HIV/AIDS in Vietnam with a high rate of HCV coinfection.

The Vietnamese Ministry of Health has recently developed national HCV Guidelines upon the release of the first World Health Organization (WHO) Guidelines on HCV care and treatment [2]. However screening and access to care and treatment for chronic hepatitis C in Vietnam are still very limited. To date, no clinical data on HCV-related liver disease are available in people living with HIV/AIDS in Vietnam and therefore the proportion of patients in need of treatment is unknown. Since new highly effective direct antiviral agents DAAs will soon be recommended by WHO as preferred options for treatment globally and are increasingly recommended by national guidelines and some of them have now become available at generic price, access to these drugs in resource-constrained areas has been urged by the medical community and the civil society [12–15].

Therefore, it is critical to identify patients in need of urgent treatment in developing countries, which bear the highest burden of HCV-related liver disease.

The following cross-sectional study aimed to assess the proportion of clinically significant fibrosis (≥F2) in HIV-HCV-coinfected patients followed in Viet Tiep Hospital in Haiphong, Northern Vietnam. We also identified epidemiological, virological and clinical characteristics associated with extensive fibrosis/cirrhosis. Since WHO [2] ranked the aspartate transaminase (AST)-to-platelet ratio index (APRI) and Fibrosis-4 score (FIB-4) as preferred non-invasive markers of fibrosis in resource-limited settings, we also evaluated the diagnostic performances of these tests in our HIV-HCV coinfected population using hepatic transient elastography (Fibroscan^®^) as a gold standard.

## Patients and Methods

### Study population

Among the 1,184 HIV-infected patients (including 1,173 (99%) under ART) who are regularly followed up in the outpatient clinic (OPC) of Viet Tiep hospital in Haiphong, we prospectively enrolled consecutive HIV-infected patients under ART with a HCV positive rapid test (SD Bioline anti-HCV rapid test (Standard Diagnostics Inc., Korea), who visited the clinic for their regular HIV follow-up between February and March 2014. As the ultimate goal of this study was to identify and characterize patients who may benefit from HCV treatment (only peg-interferon and ribavirine have been registered as HCV antiviral therapy in Vietnam), eligibility criteria for this cross-sectional study were: being over 18 years old, HCV treatment-naïve, under ART for more than 6 months with CD4 ≥ 200/mm^3^ and having no contra-indications to immediate interferon-based HCV treatment i.e: ongoing opportunistic infection, concomitant malignancy, severe anemia defined by Hb<8 g/dL, decompensated cirrhosis, acute heart failure, severe psychiatric disorders, pregnancy, platelet count <50,000/mm^3^, neutrophil count <750/mL measured in the past 6 months. Only participants with a confirmed positive HCV serology were included in the final analysis.

The study, its protocol and the written consent procedure were approved by the Ethic Committee of the Viet Tiep Hospital and the Institutional Review Board (IRB) of the Haiphong Medical Services (permit number 01BVVT/HDKH). Before enrollment in the following study, all participants provided written consents which have been kept at the ANRS site in Vietnam as recommended by the IRB. The study was carried out in strict accordance with the recommendations of the Haiphong healthcare services and the Helsinki declaration.

### Clinical assessment

Each participant was administrated a face-to-face standardized socio-demographic questionnaire in Vietnamese, had a clinical examination and a comprehensive liver assessment by a trained medical doctor. Heavy alcohol consumption was defined as a daily consumption over 50 g per day. HCV duration was estimated as “time from HCV diagnosis” defined by the duration between the discovery of HCV infection (defined by positive HCV antibody) and the time of enrolment in our study.

Liver fibrosis was assessed using transient elastography (Fibroscan 402, Echosens, France) by two trained experienced operators (TNT, DL) according to the manufacturer’s protocol [16]. The value of liver stiffness measurement (LSM) was expressed in kilopascal (kPa) as the median of 10 successful acquisitions. Unreliable measurement was defined as interquartile range (IQR)/LSM of>0.30 when LSM is ≥7.1 kPa according to Boursier et al. criteria [17]. LSM were all done fasting as recommended [18, 19]. The following cut-offs were used to stage the liver fibrosis: fibrosis F0-F1<7.1 kPa; F2: 7.1–9.4 kPa; F3: 9.5–12.4 kPa, F4> 12.4 kPa [20].

### Laboratory investigations

The following blood parameters were determined after overnight fasting in the laboratory of Viet Tiep hospital on the same day as transient elastography in all patients: AST, alanine aminotransferase (ALT), gamma-glutamyltransferase (GGT), total bilirubin, platelet count, serum albumin and prothrombin time. All participants had a confirmatory test for HCV serology by Enzyme-linked Immunosorbent essay (ELISA) technique using Phamatech anti HCV EIA kit (USA). The plasma was prepared and stored at -20°C in Viet Tiep Hospital then transferred to Pasteur Institute in Ho Chi Minh City for HIV and HCV RNA quantification and HCV genotyping. HIV and HCV RNA were extracted from plasma samples using MagNa Pure 96 system kit on the LightCycler® 480 instrument (Roche, USA). HIV viral load was measured by HIV RNA levels (Generic HIV Viral Load®, Biocentric, France) with a detection limit of 300 copies/mL [21]. HCV RNA was quantified by HCV Real-time Quant kit (Sacace Biotechnologies, Italy) with a detection limit of 15 IU/mL.

HCV genotyping was performed for samples with HCV RNA level above 1,000 copies/mL. An in-house assay was applied in which a targeting of 377 bp HCV NS5b region or 464 bp core gene was amplified by ABI 9700 PCR instrument and sequenced by CEQ 8000 Genetic Analysis System (Beckman Coulter). HCV subtypes were identified using the Los Alamos Hepatitis C sequence (www.hcv.lanl.gov).

### Statistical analysis

All data were registered anonymously in a computerized database. The description of socio-demographical, clinical, biochemical, hematological, virological and immunological parameters was performed using standard descriptive methods, i.e. categorical variables were expressed as raw numbers and percentages, continuous variables were described by their means and standard deviation (normal distribution) or their median and IQR 25–75% (non normal distribution assessed by Shapiro-Wilks test). The proportion of F3-F4 fibrosis was assessed as the number of patients with LSM above 9.4kPa and expressed with its 95% confidence interval. Mean (+/- standard derivation (SD)) score was also determined and used as a continuous variable. As transient elastography has been shown to predict portal hypertension in chronic liver disease [22] and LSM values above 20 and 40 kPa have been proved to be associated with a high risk of portal hypertension and cirrhosis-related complications, respectively [20], we also reported the number of patients with LSM >20 kPa and >40 kPa. Categorical variables were compared using a Pearson v2-test or Fisher’s Exact Test as appropriate, and continuous variables were compared using a Student’s t test or non-parametric tests as appropriate. A two-sided P value <0.05 was considered statistically significant. The diagnostic performances of APRI and FIB-4 in diagnosing advanced fibrosis(F3)/cirrhosis were evaluated from the Area Under the ROC curves (AUROCs), considering transient elastography as the gold standard. Two thresholds for fibrosis ≥ F4 with APRI were examined: 2 (high threshold) and 1 (low threshold), as per WHO hepatitis C Guidelines [2]. Likewise, two thresholds for fibrosis ≥ F3 with FIB-4 were examined: 3.25 (high threshold) and 1.25 (low threshold). The degree of correlation between both biochemical scores and result of elastometry was also explored using Spearman correlation test. Finally, risk factors for liver fibrosis ≥F3 were assessed by means of two methods: i) outcome expressed as a dichotomous variable (< or ≥ F2): univariate and multivariate logistic regression with odds ratios (OR) and 95% confidence intervals; ii) outcome expressed as a continuous variable (LSM): univariate and multivariate linear regression with β parameters and 95% confidence intervals (CI). Statistical analyses were performed using STATA 11 statistical package for Windows.

## Results

### Study population

**Fig 1:**
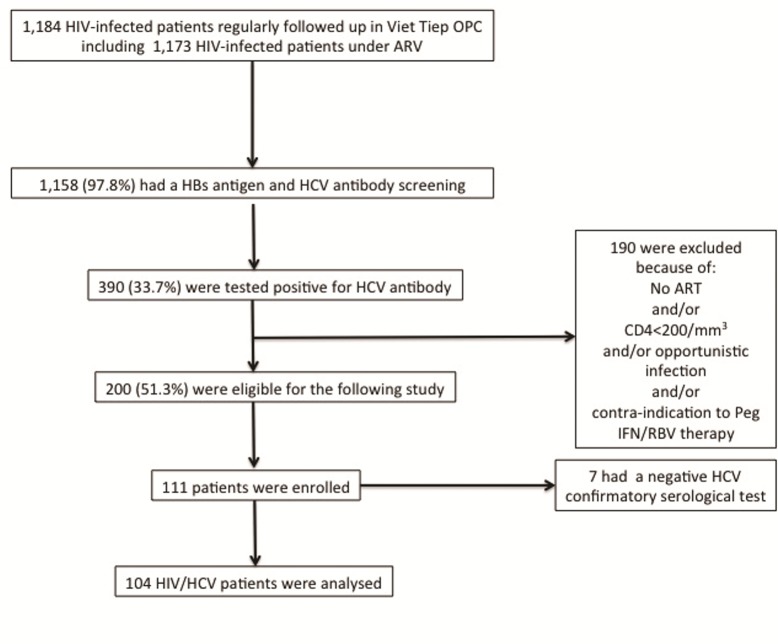
Flowchart of the study population.

Out of the 1,184 HIV patients who are regularly followed up in the OPC of the Viet Tiep hospital, 1,158 (97.8%) had a systematic screening for HBs antigen and HCV antibody and 390 (33.7%) had a positive HCV test. Of the 390 patients tested positive for HCV antibody, 200 (51.3%) were eligible for the following study. Of these 200 patients, 111 consecutive HIV and HCV co-infected adults were enrolled from February to March 2014 (Fig 1).

However, seven had a negative HCV confirmatory serological test and were excluded from further assessment. Therefore, the study population consisted of 104 patients, whose demographic, clinical and biological characteristics are summarized in Tables 1 and 2.

**Table 1:** Description of the study population according to the liver fibrosis status. Continuous variables are expressed with their median and interquartile range (IQR) for the general population and mean and standard deviation (SD) when categorized by liver fibrosis group.

	General population n = 104	Patients with F0-2 liver fibrosis n = 80	Patients with F3-4 liver fibrosis n = 24	P value
**Socio-demographic characteristics**	** **	** **	** **	** **
Male gender (n, %)	99 (95.2)	75 (93.8)	24 (100)	0.6
Age, years	35.8 (32.7–39.6)	37.1 (6.2)	36.6 (5.5)	0.9
BMI, kg/m^2^	19.6 (18.4–21.6)	20.1 (2.0)	20.1 (2.3)	0.8
IV drug use as way of HCV transmission (n, %)	72 (69.2)	56 (70)	16 (66.7)	0.8
Current or past excessive alcohol consumption (n, %)	70 (67.3)	50 (62.5)	20 (83.3)	0.04
Current tobacco use (n, %)	82 (78.9)	65 (81.3)	17 (70.8)	0.3
Current cannabis use (n, %)	16 (15.4)	11 (13.8)	5 (20.8)	0.5
Current use of any drugs (including injecting) (n, %)	8 (10.8)	6 (10.3)	2 (12.5)	0.9
Current use of injecting drugs (n, %)	9 (12.7)	7 (12.5)	2 (13.3)	0.9
Current oral opioid substitution (n, %)	21 (20.2)	18 (22.5)	3 (12.5)	0.4
**HIV characteristics**	** **	** **	** **	** **
Estimated duration of HIV infection from first screening, years	6.3 (3.1–7.8)	6.6 (4.0)	5.7 (3.8)	0.4
CD4 nadir, per mm3	84 (38–175)	113 (113.4)	133 (110.2)	0.2
Current CD4 count, per mm3	504 (361–624)	521 (210.2)	471 (159.6)	0.4
Undetectable HIV-RNA (n, %)	98 (94.2)	75 (93.8)	23 (95.8)	0.9
Duration of cART, years	4.3 (4.1–6.0)	4.2 (1.9)	3.8 (1.9)	0.3
**Type of ART**				
2 NNRTI and Nevirapine	7 (6.7)	4 (5)	3 (12.5)	0.07
2 NNRTI and Efavirenz-	91 (87.5)	73 (91.3)	18 (75.0)	0.08
2 NNRTI and Lopinavir/ritonavir	6 (5.8)	3 (3.5)	3 (12.5)	0.07
**HBsAg positivity (n, %),** n = 103	13 (12.6)	9 (11.3)	4 (12.6)	0.5

**Table 2:** Hepatitis C characteristics of the study population according to the liver fibrosis status Continuous variables are expressed with their median and interquartile range (IQR) for the general population and mean and standard deviation (SD) when categorized by liver fibrosis group.

	General population n = 104	Patients with F0-2 liver fibrosis n = 80	Patients with F3-4 liver fibrosis n = 24	P value
**Estimated time from HCV diagnosis, years**	4.8 (2.7–6.5)	4.6 (2.1)	9.0 (22.4)	0.08
**Detectable HCV-RNA, n (%)**	93 (89.4)	70 (87.5)	23 (95.8)	0.5
**HCV-RNA, Log10**	6.19 (4.95–6.83)	5.93 (1.26)	5.91 (0.99)	0.7
**HCV genotype 1**	64 (68.8)	49 (70.0)	15 (65.2)	0.9
**HCV genotype 3b**	5 (5.4)	4 (5.7)	1 (4.4)	0.8
**HCV Genotype 6**	24 (25.8)	17 (24.3)	7 (30.4)	0.6
**Platelets, 109/L**	214 (177–267)	235 (71)	189 (86)	0.003
**ALT, IU/mL**	62 (40–95)	62 (34)	128 (92)	0.0001
**AST, IU/mL**	48 (38–68)	47 (20)	105 (58)	0.0001
**GGT, IU/mL**	143 (93–378)	23.5 (210.5)	67.9 (332.5)	0.0001
**Total Bilirubin mg/dL**	10 (7–13.5)	9.9 (4.3)	16.3 (9.2)	0.0001
**Albumin, g/L**	43 (41–45)	41.8 (7,0)	41.0 (8.7)	0.9
**Prothrombin time, %**	91 (79–99)	93 (22)	79 (17)	0.002
**LSM, kPa**	6.7 (5.3–9.2)	6.1 (1.5)	18.4 (11.6)	0.0001
**APRI score**	0.7 (0.4–1.4)	0.7 (0.5)	2.2 (1.9)	0.0001
**FIB-4 score**	1.09 (0.78–1.58)	1.01 (0.77–1.25)	1.92 (1.28–1.83)	<0.0001

Study participants were mainly men (95.2%) with a median age of 35.8 (32.7–39.6) years. At enrolment, they were all under ART (most prescribed 3^rd^ agent: efavirenz with a backbone of TDF/3TC or D4T/3TC or AZT/3TC), with 94.2% rate of undetectable HIV-RNA and a median CD4 cell count of 504 (361–624) /mm^3^. The median duration from HIV diagnosis was 6.3 (3.1–7.8) years.

Of the 104 study participants, 72 (69.2%) reported past injection drug use while 24 (33%) were still injecting drugs and 21 (20.2%) were receiving opiate substitution treatment. Current or past excessive alcohol consumption was reported in 70 (67.3%) patients. The prevalence of positive HBs antigen (HBsAg) was 12.6% (95% CI: 10.4–14.8). All of the 13 HBV-infected patients except one had undetectable HBV DNA.

#### Hepatitis C and liver fibrosis evaluation

Among the 104 patients with confirmed HCV antibodies, 93 (89.4%) had a positive HCV RNA with a median viral load of 6.19 (4.95–6.83 Log_10_ IU/mL). Genotype distribution showed a highest proportion of genotypes 1 (64 (68.8%), genotype 1a and 1b: 42 (45.2%) and 22 (23.7%), respectively),followed by genotype 6 (24 (25.8%), genotype 6a and 6b: 19 (20.4%) and 5 (5.4%), respectively) and genotype 3b (5 (5.4%)). A valid LSM was obtained for all the subjects: median LSM 6.7 kPa (5.3–9.2). According to the predetermined cut-offs for liver fibrosis, 61 (68.7%) patients were classified as having no or mild fibrosis (F1),19 (18.3%) as having clinically significant fibrosis (F2), 10 (9.6%) as having extensive fibrosis (F3) and 14 (13.5%) as having cirrhosis (F4). All cirrhotic subjects had compensated liver disease and 2 were classified Child-Pugh score B. Of the 14 cirrhotic patients, 8 (57%) had LSM over 20 kPa suggesting clinically significant portal hypertension and 2 (14%) had LSM over 40 kPa, a cut-off associated with high risk of cirrhosis-related complications.

Patients with extensive fibrosis/cirrhosis had lower platelet count (P = 0.003) and lower prothrombin time (p<0.0001), and higher ALT, AST, GGT and total bilirubin levels, (P<0.0001) compared to patients with none to moderate fibrosis. A higher rate of alcohol consumption and a trend towards longer time from HCV diagnosis were also noted in patients with severe fibrosis (83.3% versus 62.5% P = 0.04 and 9 years versus 4.6 years P = 0.08, respectively). The proportion of patients with severe fibrosis was similar across HCV genotype (Table 2).

#### Risk factors for advanced fibrosis/cirrhosis

In univariate analysis with fibrosis as a dichotomous variable (F0-F2 versus F3-F4), the following factors were associated with extensive fibrosis/cirrhosis: current or past excessive alcohol consumption, HCV time from diagnosis, nevirapine or lopinavir-based ART regimen and CD4 nadir. After adjustment, the only factor remaining associated with F3-F4 score was current or past excessive alcohol consumption (OR: 4.2, 95% CI: 1.1–15.4). In multivariate analysis with transient elastography as a continuous variable, heavy alcohol consumption remained strongly associated with high LSM values (β = 36.04 (3.95–68.13), p<0.03)

#### Performance of APRI and FIB-4 scores for the diagnosis of advanced fibrosis/cirrhosis

**Fig 2:**
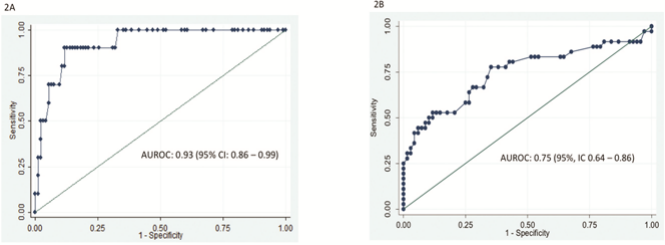
Performance of APRI in diagnosing extensive fibrosis (F3) and cirrhosis using a high threshold (APRI > 2) (Fig 1A) and a low threshold (APRI>1) (Fig 1B).

**Fig 3:**
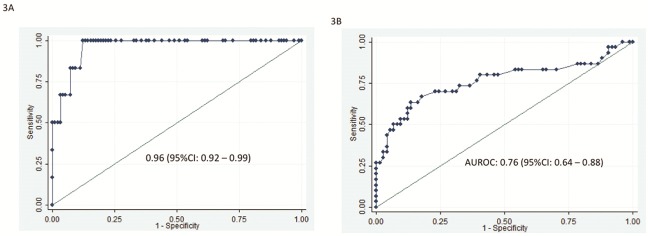
Performance of FIB-4 in diagnosing extensive fibrosis (F3) using a high threshold (FIB-4 > 3.25) (Fig 3A) and a low threshold (FIB-4 > 1.25) (Fig 3B).

The correlation between APRI and elastography scores was good (rho = 0,49, P<0,0001) and very good for FIB-4 and elastography (rho = 0,72, P<0,0001). For the diagnosis of advanced fibrosis/cirrhosis, the performance of APRI (high threshold at 2) was very good (Fig 2A and Table 3) with an AUROC at 0.93 (95% IC, 0.86–0.99) and 87 (84%) of the patients were correctly classified. Using the low APRI threshold at 1, AUROC was only 0.75 (95% IC, 0.64–0.86) (Fig 2B and less patients (78, 75%) were correctly classified. Using the high cut-off APRI score allowed maximising the sensitivity of the score (90%) while keeping the same level of specificity (84%) (Table 3). The same calculations were applied to FIB-4: for the diagnosis of advanced fibrosis (F3), the performance of FIB-4 (high threshold at 3.25) was very good (Fig 3A and Table 3) with an AUROC at 0.96 (95% IC, 0.92–0.99) and 85 (82%) of the patients were correctly classified. Using the low FIB-4 threshold at 1.25, AUROC was only 0.76 (95% IC 0.64–0.88) (Fig 3B) and less patients (82.79%) were correctly classified (Table 3)

**Table 3:** Performances of APRI and FIB-4 in predicting advanced fibrosis /cirrhosis (F3/F4) using transient elastography as a gold standard. ***Abbreviations*:** AUROC: area under the receiving operator curve; Se: sensitivity; Sp: specificity; LR-: negative likelihood ratio; LR+: positive likelihood ratio.

	AUROC (95%CI)	Se (%)	Sp (%)	Correctly classified, (%)	LR-	LR+
High APRI threshold (2)	0.93 (0.86–0.99)	90	84	84.6	0.11	7.69
Low APRI threshold (1)	0.75 (0.64–0.86)	47	90	75	0.59	4.59
High FIB-4 threshold (3.25)	0.96 (0.92–0.99)	100	81	81.9	0	5.96
Low FIB-4 threshold (1.25)	0.76 (0.64–0.88)	53	88	77.9	0.53	4.39

## Discussion

Our study suggests that among HIV-HCV coinfected subjects under ART who are attending the outpatient clinic in Haiphong, Northern Vietnam, almost half of them have clinically significant fibrosis (≥F2) including one fourth with extensive fibrosis or cirrhosis despite optimal HIV viro-immunological control. We confirm that genotypes 1 and 6 are the predominant HCV genotypes circulating among this group but in different proportions than in Hai Phong and other regions of Vietnam.

Few studies on HCV infection have been conducted in Vietnam. These studies were mainly sero-epidemiological surveys without clinical evaluation of the liver disease [9, 23, 24]. In Southeast Asia, only one study from Thailand assessed the degree of liver fibrosis in 130 HIV-HCV co-infected individuals [25]. This study reported 41.7% of HIV-HCV coinfected patients with extensive fibrosis F3/F4 using Fibroscan^®^. Out of them 24% were classified as cirrhotics. Cross-sectional studies conducted in Western countries also reported high proportions of extensive fibrosis and cirrhosis in cohorts of HCV naïve of treatment HIV-HCV coinfected subjects [26–29]. The higher rates of extensive fibrosis observed in the Thai and Western studies compared to our results may be explained by the differences in age (our patients were much younger) and estimated time from HCV infection. The median duration of HCV exposure was over 17 years in the Thai study and the Western studies as compared to an estimated median HCV contamination below seven years in our study.

Interestingly, the short period from HCV diagnosis (4.8 (2.7–6.5) years) suggests that HCV infection was long neglected in Vietnam, as systematic HCV screening has been implemented only in 2005 in HIV clinics.

Haiphong city experiences important rates of injection drug use in young individuals [30]. Indeed, it is a well-established area within the “Golden Triangle” for heroin supply at low price and has become an attractive area for PWID [30]. In our study, as expected the use of injecting drugs was frequently associated with HCV infection among HIV positive patients and HCV transmission was attributable to the use of intravenous drug in almost two third of the cases. This is in line with other studies from Vietnam, and other Asian countries that reported a high prevalence of HCV infection, up to 96% in injecting drug users [9, 10, 31]. In Hanoi, among HIV-infected individuals, 35.4% were tested positive for HCV and HIV-HCV coinfection was mainly observed in young males. In this study, the use of injecting drug was reported as a transmission factor in 44% of cases [23].

As suggested by previous studies, we confirm that genotypes 1 and 6 are the most frequent circulating genotypes in Vietnam [32, 33]. However, genotype 6 was not the predominant circulating genotype in our study population although previous studies reported a higher prevalence of HCV genotype 6 in Vietnam [32, 33].

While Avihingsanon et al. observed a lower rate of severe fibrosis in HCV genotype 6-infected subjects in Thailand, we did not find any difference in fibrosis stage according to genotype.

We assessed the severity of liver fibrosis using Fibroscan^®^ as its excellent performances have been demonstrated in HCV-infected as well as HIV-HCV co-infected individuals [20, 34, 35]. In addition, liver biopsy is not feasible in routine clinical practice in resource-limited countries. However, the Fibroscan^®^ device is rarely available in these countries mainly because of its high cost (€34,000 for the portable machine and €5,000 for the annual maintenance cost). In Vietnam only a few machines are available in the country and are mainly accessible in the private sector. As a result the WHO recommends the use of APRI or FIB-4 as simple and inexpensive alternative methods to identify patients with extensive fibrosis and cirrhosis in resource-constrained countries [2]. Using Fibroscan^®^ as a gold standard, we found that APRI and FIB-4 performed very well in diagnosing extensive fibrosis/cirrhosis (AUROC>0.90) when using the high thresholds of 2 for APRI and 3.25 for FIB-4.

Since international guidelines recommend to prioritize treatment in patients with extensive fibrosis or cirrhosis [36], we focused our analysis on F3-F4 patients. A recent model-based analysis performed in resource-limited countries (Egypt, Thailand, Côte d’Ivoire) also suggested that focusing HCV treatment on patients with severe fibrosis (F3–F4) using standard of care therapy (Peg-interferon and ribavirin) or interferon-free therapy (sofosbuvir and ribavirin), is the best strategy for developing countries as it provides the highest number of life per year saved [37]. Several studies have also shown that HCV antiviral therapy in IDUs, especially in those on opioid substitution treatment, is associated to identical sustained virological response compared to non-or former IDUs [38, 39]. In addition, there is an increase evidence to propose HCV therapy as a cost-effective intervention to reduce the burden of HCV infection and transmission in this specific population [40, 41].

Our study clearly suggests that a high proportion of IDUs in Vietnam urgently requires HCV antiviral therapy. Based on our analysis and considering that there is 200,000 IDUs in Vietnam, about 150,000 of them (70%) have been infected with HCV, 80% of them (120,000) have a chronic HCV hepatitis and at least 30,000 could be pre-cirrhotic or cirrhotic with a risk of hepatic decompensation or HCC over the next years if they do not benefit of antiviral treatment soon.

Interestingly, almost all our study participants (95%) had a good HIV viro-immunological control. This reflects the great efforts that have been deployed over the last decades in Vietnam to fight against HIV/AIDS and expand ART also to the most stigmatized populations. Thus, if access to HCV treatment is not improved, the benefits of ART on mortality will be clearly undermined.

In our study, heavy alcohol consumption was the only statistical difference between patients with extensive fibrosis or cirrhosis and those with none to moderate fibrosis. It was also a strong independent factor of severe fibrosis. By contrast with other studies, age, estimated duration of HIV or HCV infection and ARV regimen were not statistically associated with extensive liver fibrosis/cirrhosis although a trend was noted for the HCV duration. This underlines that excessive alcohol consumption is an important contributor of liver fibrosis progression in this population. Heavy alcohol consumption has been previously reported as a major public health problem in Vietnam in particular in men [4, 42]. Unfortunately, alcohol is another neglected problem in many resource-limited countries and must be clearly integrated in harm reduction programmes [2].

Our study has some limitations. First, it is a cross-sectional study including a small number of patients, with a reduced statistical power to assess risk factors, which may not strictly reflect the burden of HCV-related burden disease in HIV-HCV individuals in Vietnam. Secondly, since the most severe cases have been seen mainly in inpatient clinic, our study may have underestimated the proportion of patients with end-stage liver disease. Thirdly, we assessed the diagnostic performances of biochemical markers of fibrosis using transient elastography as a gold standard although we could not validate its performances against liver biopsy in our study population. However, excellent performances of Fibroscan in predicting liver fibrosis in Asian HCV subjects have been previously reported using liver histology as a gold standard [43]. Finally, we could not properly assess the proportion of patients with liver complications (in particular portal hypertension) due to resource-limited constraints.

Our findings underline the urgent need of HCV “screen and treat” intervention programmes in Vietnam. Among HIV-HCV coinfected subjects successfully treated with ART, our study suggests that about 25% of patients urgently require HCV antiviral therapy. This underlines the necessity to rapidly expand access to HCV treatments adapted to the local epidemiology (genotypes 1 & 6). This will comply with the recent WHO recommendations [2] and its urgent call to deploy strategies to reduce the burden of viral hepatitis especially in endemic resource-constrained areas [44] and eventually eliminate the epidemic globally [45].
